# A Lecture, Introductory to the General Course in the Chicago Medical College, for the Session of 1870–71

**Published:** 1870-11

**Authors:** H. A. Johnson

**Affiliations:** Professor of Diseases of the Respiratory and Circulatory Organs


CHICAGO MEDICAL EXAMINER.

N. S. DAVIS, M.D., Editor.

VOL. XI. NOVEMBER, 1870. NO. 11.

### Original Contributions.

ARTICLE XXXIV.

### A LECTURE, INTRODUCTORY TO THE GENERAL COURSE IN THE CHICAGO MEDICAL COLLEGE, FOR THE SESSION OF 1870–71.

By H. A. JOHNSON, M.D.,
Professor of Diseases of the Respiratory and Circulatory Organs.

What is meant by the study of medicine? How should it be pursued? What helps do medical colleges generally afford? What are the peculiar aids afforded by this institution? Such, gentlemen, are the questions that must have engaged your thoughts heretofore, and that, almost of necessity, present themselves on this occasion.

### WHAT IS THE STUDY OF MEDICINE?

In answer to the first question, it may be stated, in brief, that medicine, in the more general sense of the term, is made up of pure science, and applied science, or art. It is broad in its relations, and, for its apprehension, an important prerequisite is a good, liberal, scientific, and, if possible, literary education. The success or failure of men in the profession depends much more upon this general preparation for special work than is usually supposed. By the study of medicine, we mean, first, the study of those sciences that have to do with the human organism; the structure, chemical composition, and uses, of its parts, together with the forces, both physical and those, which, for want of a better term, we call vital, that seem to belong to its forms; or, in the words of a modern formula, the modes of motion therein observed. To these subjective studies must be added a careful investigation of all those influences, or agents, that, acting from without, impress, for good or evil, the mechanism of life. This first department of study embraces anatomy, physiology, chemistry—inorganic and organic, general pathology, etiology, hygiene, the general principles of cure, and the natural history and properties of drugs. The second department of study consists in the application of the facts embraced in this first group. In other words, it is applied science. It includes the study of special diseases, with reference to their cure, or relief, by the control or modification of the modes of motion within the body, or of influences acting upon it from without. It is made up of what are usually termed the practical branches, or the principles and practice of medicine, surgery, and obstetrics—the principles usually taught in didactic lectures; the practice best taught at the bedside of the patient.

HOW SHOULD THIS STUDY BE PURSUED?

From this brief statement of the subjects of study, it must be evident to you, gentlemen, that, in the order of time, the facts must be learned first; afterwards, their application. The more distinctly scientific studies must precede those which are mixed in their character (being both science and art), and which often present problems more difficult than any which are found in the domain of purely physical science. That they so frequently remain unsolved is, because our knowledge of the precedent facts is so limited.

The best method, or rather order, of study suggested by this examination of the subjects to be investigated, is a matter, gentlemen, of the utmost importance. The student of Greek would hardly attempt to write Greek poetry without having first studied Greek roots, and thoroughly mastered the grammar of the language. One may *guess*, perhaps, the solution of a problem in applied mathematics, but he will not be likely to know much about it, unless he has first, by patient study, possessed himself of the fundamental principles of the science. The architect may have some idea of beauty of proportions and fitness of forms, but cannot, safely, be trusted to design and superintend the erection, or repairs, of an important structure, unless he has first learned the weight and strength of his material, the direction and quantity of resultant forces, with many other facts in physical science that are directly concerned in his art-work. The order of study, in these supposed cases, is progressive, each subject occupying, in the order of time, the position, which, in the order of science, it logically sustains to every other subject of investigation. There is no good reason why medical studies should not be pursued in the same way; in fact, such is the case now everywhere, except in our own country.

WHAT AID DO THE COLLEGES GENERALLY AFFORD?

The colleges organized anterior to the war of independence were, for the most part, under the control of men who had been thoroughly educated in the schools of Europe. The preliminary and professional attainments required were of a high order, and the degree of doctor in medicine conferred upon only the very few who had afforded the most satisfactory evidences of proficiency. These schools were intended to *educate* the pupil—to discipline his faculties, as well as to enrich his memory; to develope his powers of reasoning, and to prepare him to successfully grapple with the problems of practical therapeutics.

The number of pupils, however, in attendance, was limited. The larger majority of physicians were unable, or unwilling, to avail themselves of the advantages offered by these institutions located in New York and Philadelphia.

In most instances, the education of the pupil was received in the office of the preceptor, who directed the subjects and order of study, and, after the acquisition of a limited knowledge of the elementary branches, permitted him to see cases, and, finally, to prescribe independently, or, in other words, admitted him to practice. This education was, necessarily, sadly deficient, for the want of the means of illustration, and, in too many instances, from the incompetence or indolence of the instructor.

At the end of three or four years, however, the student had obtained, at least, a superficial knowledge of most of the subjects at that time embraced in the curricula of the medical schools of the Old World. The first medical schools established in this country, subsequent to the war of independence, seem to have had quite another purpose. They were intended simply to supply the deficiencies necessarily connected with private teaching. Their germs, so to speak, consisted of lectures on anatomy, to which were subsequently added brief courses on other departments of study, until the college was finally developed. They were intended quite as much as, or, perhaps, more for the practitioner than for the student. The courses were short, usually only thirteen weeks, and the whole field of medicine, surgery, and obstetrics, was passed rapidly in review, rather than carefully considered and taught in detail. The *studies* of the pupil were still supposed to be performed in the office of the preceptor, and the college resorted to for the purpose of refreshing the memory.

It is true, students were received at the very commencement of their period of study, but such usually went out after one course of lectures, and commenced practice, occasionally returning, a few years later, to listen to a repetition of the same course, and receive the degree. The gray-haired veteran, who knew almost nothing of the science of medicine, and the beardless boy, who knew absolutely nothing of the practice, sat side by side, and listened—the one laboriously endeavoring to correct his previous mistakes by placing a foundation beneath his shabby building, and the other, with still more embarrassment, if possible, striving to erect, simultaneously, both foundation and superstructnre. During this present century, great changes have been made in our material, intellectual, and social condition. Higher attainments are demanded in all the professions, but especially in that of medicine. Contributions have been brought to it from every department of science. It has been continuously enriched by new truths. By a judicious division of labor, men peculiarly adapted to special studies and practice, have improved its methods and corrected its fallacies. The cords of its tent have been lengthened, and its stakes strengthened; but, with few exceptions, the colleges are to-day what they were in the infancy of the Republic. Amid all these changes in other departments of education, they alone seem to say *non possumus*. It is true, some of them have extended the terms of lectures; a very few, only, have increased the number of professorships, and have added to their means of illustration, but the system is still based upon the hypothesis—at the present day, only an hypothesis, that the college-lectures are simply a review of what has already been made the subjects of study in the office, or that the pupil is getting a hasty and imperfect preparation for a period of practice, after which he will return and refresh his memory.

It is possible, that in the last quarter of the eighteenth century, when but few could spend the time or means necessary to a thorough preparation, but when many could devote a few weeks to a hasty review, this method of college instruction might have been admissible; but, in the last half of the nineteenth century, when physicians, receiving pupils into their office, assume little responsibility for their studies, seldom directing their reading, or, in fact, doing anything more than to furnish a few antiquated text-books, and, perhaps, a dilapidated skeleton, while the student, if not very conscientious, amuses himself by reading what taxes the least his mental digestion, whether it be "Gray's Anatomy of the Human Body," or "Burton's Anatomy of Melancholy,"—pathological anatomy, or pathological psychology—always looking forward to the college as his educator, and considering the office as a cheap and convenient, if not agreeable, sojourn for the required three years; in this age, and in this country, where we especially boast of our improved methods of education—where our public schools are graded—where, in our literary and scientific institutions of a higher order, the pupil advances step by step—where, in our professional schools, legal and theological, there is method in the arrangement of studies;—under all these circumstances, with all this new condition of things around us, this inertia of the medical schools—their persistance in the attempt to teach the same truths to those commencing, and those completing, their education; to the tyro just learning to pronounce his anatomical alphabet, and to the applicant for the doctorate, is worse than an anomaly—is worse than an absurdity even; inasmuch as it encourages a contentment with superficial attainments, thereby diminishing the proficiency of the profession, it is a crime against humanity.

These defects in our methods of education have been recognized by the profession, and, for the last thirty years, medical societies and prominent medical men have protested against them. The American Medical Association has repeatedly called for reforms, and the faculties of the colleges have almost unanimously admitted the justness of the complaints and the propriety of some change in the curriculum of study. There is almost no one to defend the old order of things, and yet it continues. Why it continues is no mystery to medical teachers. The principal reason is, undoubtedly, the competition between the different medical schools. It is said that students will attend such colleges as make their admission to the profession easy—as require the least work and the shortest time; and, for this reason, no one institution had, up to 1859, dared to establish a progressive course of study. In illustration of the general feeling on this subject, I take the liberty of citing a portion of the history of one of the institutions of our own city. In the year 1857, the faculty, by, if I remember rightly, a unanimous vote of all present, (one member only being absent), decided to adopt a curriculum of study, extending through two years, dividing the subjects into two departments, to be called junior and senior, and requiring students attending their first course of lectures to enter the junior department, made up of the elementary branches, and those attending their second course of lectures to enter the senior department, composed of the practical branches. The adoption of this plan by a formal vote of the Faculty, after mature deliberation, was, in itself, a recognition of the imperfections and inconsistencies of a system, that whatever might have been its utility at an earlier period in our history, had become effete. This wise measure, however, was never put into operation. The President of the College admitted the propriety of the change, believed that it would make better physicians, but expressed a fear that students would be repelled by the increased labor imposed and higher attainments exacted. In deference to this apprehension, without any reconsideration of the vote, the old method was continued, and no improvement has been made, I believe, up to the present time. Other medical teachers privately expressed themselves as heartily tired of the prevailing system, if indeed it be a system, but were restrained from attempting reforms by the same considerations—a fear that their pupils would seek for their diplomas in competing schools, where the labor was less, and the standard consequently, lower. In the conventions of medical colleges that have been held in connection with the American Medical Association, there has not been a single reason urged for the existing order of things. The only apology offered in its defence is, that it has made good physicians. The fact is, the schools do not make physicians, but they help individuals, more or less, to qualify themselves for the office, and the better the help, the easier the qualification is obtained. Men have become good mechanics without masters, able lawyers without instructors, eloquent divines without professors, and skilful physicians without preceptors, but these examples by no means constitute a reason why the best aids to excellence in these several departments of effort should be ignored or neglected. Notwithstanding a very general admission of failures by those interested in teaching, no action has been had, because each school feared that its neighbor would not come up to the standard. The members of the profession at large, represented in the National Association, have, from year to year, adopted reports and resolutions, all calling loudly for a change, but they have been unable to convince the colleges that medical students were equally desirous for extended courses and classified studies. We have thus briefly alluded to the history of medical schools, and to the reasons for the anomaly they present, in order to answer the second query. It must be evident to you that the colleges generally can help you by furnishing, to a limited extent, the opportunity for study, and the means of illustration, leaving the question of the arrangement of subjects entirely to yourselves. So far as physical digestion is concerned, it is not, perhaps, a matter of much consequence, whether fruits and desserts are taken before or after beefsteak and potatoes; but, so far as mental assimilation is concerned, the order in which the subjects are presented becomes a matter of the greatest importance. The accumulation, in a desultory manner, of unclassified facts, is almost as useless as a miscellaneous unclassified collection of objects in natural history. The method generally pursued is faulty, not only in this want of order, but, also, in the effort to accomplish the impossible feat of teaching the whole of the science and the art of medicine, surgery, and obstetrics, in the short period appropriated to a single lecture-term. It may be said, in answer to this, that all the colleges require three years of study and two courses of lectures. This is true, but the second course of lectures is only a repetition of the first, and the whole field is usually looked at, a kind of bird's-eye view, in from four to five months, the student gaining some knowledge of many things, but an accurate knowledge of hardly any one thing.

WHAT ARE THE PECULIAR AIDS AFFORDED BY THIS INSTITUTION?

Convinced that something better was possible, and that at least a few medical students were ready to work for a higher standard of attainments, the founders of the Chicago Medical College unanimously determined to make an earnest effort for reform.

In the Spring of 1859, it became evident that a new medical college was about to be organized in this City, and it was possible for a few individuals to control the movement, and give direction and character to the new institution.

My own connection with the Rush Medical College had recently terminated; Professors Andrews and Hollister, who had also been connected with that institution, had previously resigned. Our experience as teachers, as well as our remembrance of our own student life, had led us to the firm conviction, that these radical evils of the schools were not an absolute necessity, and we believed that the public and the profession would second our efforts for something better.

The peculiar features and relations of the Chicago Medical College must be my apology for detaining you a few moments, while I present the most important points in its history. In doing so, I shall be able to answer satisfactorily, I trust, the last of the four questions that we have proposed to ourselves this evening.

The first entry in the record book of the Faculty is as follows: "At a meeting held March 12, 1859, at the office of Drs. Rutter and Isham, the following named gentleman were present: David Rutter, M.D., Hosmer A. Johnson, M.D., Edmund Andrews, M.D., and Ralph N. Isham, M.D. It was announced in a few words by Dr. Johnson, that the object of the meeting was to organize a Medical Faculty of the Lind University, on the basis of a proposition made by the Trustees of Lind University, and submitted to the above-named gentlemen for their consideration." After a temporary organization, the proposition was considered, discussed, and finally accepted. The provisions of the agreement between the above-named and the Trustees of the University, were substantiaally as follows:

1st. That the University should furnish, for the temporary use of the Department of Medicine, rooms free of rent for three years.

2d. That at the end of three years, the University should provide a permanent building, or rooms suitable for the Department.

3d. The Faculty of Medicine to nominate, the Trustees to appoint, all professors in the Department.

4th. The Medical Professors to give the proceeds of their lectures for three years, for the purpose of procuring the means of illustration.

5th. The Faculty to meet all expenses, except that of rooms.

6th. The degrees to be conferred by the University, upon the recommendation of the Faculty.

The 7th and 8th sections relate to announcements, and provide for a report of the Treasurer of the Faculty to the Board of Trustees.

This proposition or agreement was signed, on the part of the University, by its Executive Committee, and by Drs. Rutter, Andrews, Isham, and Johnson.

At the same meeting, after the execution of the agreement, it was moved that the following professorships be established, *viz.:* Descriptive Anatomy, Physiology and Histology, Inorganic Chemistry, Materia Medica and General Therapeutics, General Pathology and Public Hygiene, Surgical Anatomy and the Operations of Surgery, Organic Chemistry and Toxicology, Medical Jurisprudence, Midwifery and the Diseases of Women and Children, Principles and Practice of Surgery, and the Principles and Practice of Medicine.

Dr. Rutter was nominated for Emerites Professor of Midwifery and Diseases of Women and Children; Dr. Johnson for the Chair of Physiology and Histology, but afterwards transferred to that of Materia Medica and General Therapeutics; Dr. Andrews to the Chair of the Principles and Practice of Surgery, and Dr. Isham to the Chair of Surgical Anatomy and the Operations of Surgery.

Drs. Johnson, Andrews, and Isham were appointed a Committee to invite Prof. N. S. Davis to accept the Chair of the Principles and Practice of Medicine, and Dr. W. H. Byford to accept that of Midwifery and the Diseases of Women and Children.

These gentlemen accepted the appointments, and subsequently participated in the complete organization of the College.

The two departments, Junior and Senior, were established, the first including Descriptive Anatomy, Pysiology and Histology, Inorganic Chemistry, Materia Medica and Therapeutics, and General Pathology and Public Hygiene; the second including the remaining branches, namely, Surgical Anatomy and the Operations of Surgery, Principles and Practice of Surgery, Obstetrics and Diseases of Women and Children, Practical Medicine, Medical Jurisprudence, and Organic Chemistry and Toxicology, with Clinical Medicine and Clinical Surgery.

These two courses were intended for students at different periods of their pupilage, and the arrangement of studies was such as to prepare the pupils in the first course, for the more practical branches of the second. This classification of the subjects of medical study, was, so far as I am aware, the first attempt in the United States, to make medical teaching conform to the methods that obtain in all other departments of education at home, as well as in all medical schools abroad.

A lecture term of five months was established. The fees were fixed at fifty dollars for each course, and examinations were required at the end of the first course, and this when the lecture term of the Rush College was sixteen weeks, and the fees thirty-five dollars. The Faculty commenced with the determination, that their inducements to students, should consist in neither short terms, nor a low standard of attainments, nor comparatively, in low fees even, but rather in their better methods and more extended range of studies. They considered medicine as a liberal profession, and they determined to strive for the broadest and most liberal culture on the part of those who should come to them for instruction.

They did not expect large classes, and were determined to make no sacrifice of principle to obtain them; but they did believe and know, that they if did not send out many graduates, those that should go forth from their halls, would be more thoroughly prepared for the work of life. How these expectations have been met, the many alumni of the College holding prominent social and professional positions, bear testimony.

The organization of the Faculty was completed by the appointment, subsequently, of Dr. J. H. Hollister to the professorship of Physiology and Histology; Dr. Mahla to the two professorships of Chemistry; Dr. M. K. Taylor to the professorship of General Pathology and Public Hygiene; Dr. Titus Deville to the professorship of Descriptive Anatomy, and H. G. Spafford, Esq., to the professorship of Medical Jurisprudence. Dr. Horace Wardner was appointed Demonstrator of Anatomy.

At a meeting of the Faculty, June 4, 1859, it was resolved, that the Faculty, through their delegates, invite the Illinois

State Medical Society to appoint a committee of two to attend the examinations of candidates for graduation in the Medical Department of the University, and *vote* upon their qualification for the degree of Doctor of Medicine. In extending this invitation, the College desired to furnish to the profession at large the means of judging of the success or failure of the method, and also to give the fullest guarantees, that with the sanction of this institution, none should be admitted to this responsible office, whose attainments were not satisfactory to those not immediately interested in teaching. This invitation was continued from year to year, but the State Society, either never made the appointment, or, if so, the committee has failed to respond.

In connection with the first course of didactic lectures, the Faculty established two Clinical professorships, to which Professors Davis and Andrews were appointed, securing to the student one lecture daily in the Mercy Hospital, at the bedside of the patient, and, also, two cliniques each week at the College, thus providing a more extended course of practical teaching than had ever before been given in the West.

The class attending this first session was small, consisting of but 33 members, of whom 9 received at the commencement exercises, the degree of Doctor of Medicine.

The Faculty had entered upon this experiment, for in one sense it was an experiment, with a firm conviction that it was the right course to pursue, they were satisfied also, that ultimately the schools must adapt themselves to the increasing intelligence and higher standards of education demanded by the community; they had, however, some misgivings as to the readiness of young men to devote to this work of preparation the increased time, and necessary expense. Among the class in attendance upon this first course of lectures, however, there was a larger proportion than at that time were usually found in medical schools, of young men thoroughly prepared by scientific and classical attainments for professional study. It was evident, then, that the better quality of students, sought, what every educated man, whose interests do not blind his judgment, admits to be the better methods.

The Faculty, therefore, were quite willing to labor and to wait.

Before the commencement of the second course of lectures, Prof. Deville, a most admirable teacher of Anatomy, resigned his professorship and returned to England. Prof. Hollister was transferred to the vacant chair, Prof. Johnson was appointed to the Chair of Physiology and Histology, and Dr. A. L. McArthur was appointed to the Chair of Materia Medica.

On the 24th of November, 1861, the Trustees requested the Faculty to release them from any obligations to furnish new rooms for the Department, but agreed to pay the rent of those they then occupied in Lind's Block for the remainder of the three years from date of the organization, as stipulated in their contract with the Medical Faculty. The financial embarrassment of the University, made this action of the Trustees, a necessity, but it was the occasion of much anxiety to the friends of the College. The Faculty, however, were not disposed to abandon their projects, but immediately made arrangements to provide for themselves.

In the Summer of 1862, Dr. McArthur resigned the professorship of Materia Medica. Prof. Taylor requested leave of absence for the coming Winter, having been appointed Surgeon of Volunteers in the U. S. Army. At his own request, Prof. Hollister was transferred to the Chair of Materia Medica, and Dr. J. S. Jewell, a graduate of the College at its first commencement, appointed to the Chair of Anatomy, and also made Demonstrator of Anatomy, Dr. Henry Wing was requested to give the course on General Pathology, in the absence of Prof. Taylor, and at the end of the course was appointed Professor.

The College continued to occupy the rooms in Lind's Block, on Market Street, during the session of 1862–3, paying to the proprietors the rent out of the College funds. In the Summer of 1863, the Faculty, by a unanimons vote, resolved to appropriate the proceeds of their lectures to a building fund, for the purpose of securing new accommodations for the institution. A contract was made with C. Follansbee, Esq., for the lot and building on State Street, recently occupied by the College. By the liberality of one its members, the Faculty was subsequently entirely relieved from the indebtedness thus contracted. During this summer, Dr. M. O. Heydock was appointed to the professorship of Medical Jurisprudence, Prof. Spafford having resigned.

In the Spring of 1864, it seemed to the Faculty desirable, to change to some extent at least, their relation to the University. By a mutual understanding with the Trustees, each party to the contract was released from all obligations, and the members of the Faculty organized under the general incorporation laws of the State of Illinois, the Chicago Medical College, the members of the Faculty becoming the Trustees of the new body.

During this year some changes were made in the assignment of Chairs, Prof. Hollister was transferred to the Chair of Physiology and Histology, Prof. Wing to the Chair of Materia Medica, Prof. Johnson to the Chair of General Pathology and Public Hygiene.

In the Summer of 1865, Prof. Wing having removed from the City, was transferred to the Chair of Medical Jurisprudence, and Prof. Heydock to the Chair of Materia Medica and Therapeutics, and Dr. J. M. Woodworth was appointed Demonstrator of Anatomy.

During the year 1866, Dr. Daniel T. Nelson was appointed Professor of Physiology and Histology, Prof. Hollister was transferred to the Chair of General Pathology and Public Hygiene, Prof. Johnson having resigned, on account of ill-health, Prof. Patterson was appointed to the Chair of Medical Jurisprudence, made vacant by the resignation of Prof. Wing. In 1867, Prof. Mahla resigned the professorship of Chemistry, and Dr. John E. Davies was appointed to the vacancy. The Trustees created a new Chair, of Diseases of the Respiratory and Circulatory Organs, to which Prof. Johnson was appointed.

In May, 1867, there was held in the City of Cincinnati, a convention of delegates from the medical schools of the country: resolutions were passed recommending changes in the methods of study, substantially the same as those already adopted by this College; but advising four years of study, instead of three.

These propositions, no doubt, faithfully represented the opinions of those teachers when at a distance from their institutions, but they had altogether a different set of ideas, when the question was presented in its financial aspects, at home.

At a meeting held April 25, 1868, this Faculty arranged three courses of lectures consisting of groups of subjects or studies, for each of the three years of pupilage. Up to the present time, however, while recommending the student to attend one course during each year, it has not been found practicable to *require* this as a condition for graduation.

These three courses of lectures, not repetitions, the one of the other, but separate, distinct, and successive, constitute a more complete and extended curriculum of study, than that adopted by any other college in the United States.

During the year 1868, Prof. Davies resigned the Chairs of Chemistry, and Dr. C. Gilbert Wheeler was appointed to the position thus made vacant. The late Dr. Joseph L. Hildreth was invited to deliver a course of lectures on Ophthalmology and Otology, the Chair of Public Hygiene was detached from that of General Pathology, and Dr. Thomas Bevan appointed Professor.

During the year 1869, changes were also made in the Faculty, Prof. Jewell having resigned, Dr. H. W. Boyd was appointed Professor of Descriptive Anatomy; Dr. E. O. F. Roler was appointed an additional Professor of Obstetrics and Diseases of Women and Children; Dr. J. S. Sherman was appointed Adjunct Professor of the Principles and Practice of Surgery; Dr. Woodworth resigned the position of Demonstrator of Anatomy, and Dr. Thomas Bond was appointed to that office.

During the same year, an arrangement, mutually satisfactory, was made with the Northwestern University, by which the Chicago Medical College, became the Medical Department of that institution, its own organization, control of professorships, and of the curriculum of study remaining unchanged. The ground occupied by the present building, belonging to the proprietors of the Mercy Hospital, had been previously secured for the use of the College. By the terms of the contract between the

Trustees and Hospital authorities, the Faculty of this Institution became the medical officers of the Hospital, and are required to give in its wards, such clinical instruction as the interests of the College demand; the services of these medical officers to be considered as payment in full for the ground-rent of these premises, for at least twenty years, and so much longer as the adjacent building shall be occupied for hospital purposes. It is believed that the interests of both parties to this contract, is met in the permanent relations thus established. The material result of these arrangements with the University, and the Society owning and controlling the Hospital, is the elegant and convenient building, in which we are to-night, for the first time, assembled. It is not only architecturally attractive, and admirably adapted to medical teaching, but its location and relations to the Hospital are such, that for all practical purposes, that institution becomes a part of the college organization. Each Didactic Chair has its corresponding Clinical Chair in the Hospital, where the theories of the lecture-room are daily tested at the bedside of the patient. In this respect, also, it differs materially from most medical schools of this country, and, to the same extent, approximates in its means, as well as in its modes of teaching, the best institutions of the old world.

During the present year, the corps of instructors has been still further increased; Prof. Wheeler has resigned the two professorships of Chemistry, and the Trustees have appointed Dr. N. Gray Bartlett to the Chair of Inorganic and Practical Chemistry, and Dr. H. P. Merriman to the Chair of Organic Chemistry and Toxicology. In consequence of the large and increasing number of pupils engaged in the study of analytical chemistry, this division of the work had become absolutely necessary. A Chair of Diseases of the Eye and Ear has been definitely established, and Dr. Samuel J. Jones appointed Professor. Prof. Jones is now in Europe, or on his way home, and, with all desirable means of illustrating his department, will commence his course on the first of November. Prof. M. O. Heydock has resigned the professorship of Materia Medica and Therapeutics, and Dr. Wm. E. Quine has been appointed to the vacancy.

Since the organization of the College, eight hundred and eighty-nine (889) students have been in attendance upon its lectures, and two hundred and eighty-nine (289) have received the degree of Doctor of Medicine. Six of its Alumni are now members of its own Faculty, and several hold similar positions in other institutions.

From the period of its inception to the present, it has been both growing and developing. For the last eighty years, other medical schools have only grown. Almost nothing has been added to the number or perfection of their organs. In their relation to the times, and the intelligence of medical students, they are sadly out of joint—a kind of compound dislocation. In the number of teachers, and breadth of study, hardly a change has been made. The increase in the lecture-term, from three to four or five months, constituting, with very few exceptions, the only improvement during the last three-quarters of a century.

At the time of the organization of this College, the number of lectures, comprising the entire course of instruction in the Rush Medical College (and I refer to it because it is a fair representative of the medical colleges of the country) was five hundred and seventy-six (576). The number in the entire course of this college was at the same time one thousand and eight (1008). At the present time, the number constituting the entire course of medical instruction in the Rush Medical College, remains substantially the same, while the entire course in this institution is thirteen hundred and eighty (1380), an increase of three hundred and seventy-two (372) lectures. In other words, the amount of teaching given by the Faculty of this College is about two and one-half ($2\frac{1}{2}$) times as much as that given by the majority of the medical schools of the country, while in the enlargement of its corps of teachers, and the still further classification of the subjects of study, it has spared no effort to adapt itself to the constantly increasing culture and higher scientific attainments of the times.

We have thus rapidly traced the history of this school for the purpose of showing to you, gentlemen, that it can help you in your work of education more than other colleges by presenting to you, first, more truths and more facts, and, second, by presenting them in a better order—not, as the printer would say, a mass of type set in "pi," but set in up form, corrected, and ready to give a perfect impression.

In view of all these facts, we think we may justly claim for this College the confidence of the public and the profession. Its Faculty and Trustees have earnestly labored for the honor and usefulness of our noble calling. They have not been deterred by the ridicule or misrepresentations of those who could not appreciate honest effort, and to-day, entering as they do upon a new era in their history, one so full of encouraging promise and comforting vindication, with a better knowledge of the wants of students, and of the best methods at home and abroad, with enlarged appliances and means of illustration, affording opportunities for clinical study and for experience in practical chemistry unsurpassed anywhere upon this continent; they offer to you, gentlemen, a cordial welcome. Welcome to these halls where science shall place a sure foundation for the temple of your art. Welcome to all that we can do for you as teachers, as counsellors, as friends. Welcome, also, to yonder Hospital, where you may study the perturbed motions of the soul's most wondrous engine—where your hearts may be touched with sympathy as you listen to the wail of its broken harp, and learn how most surely and tenderly you may repair its disordered mechanism—tune again its discordant strings, and fill all the halls of being with the sweet harmonies of healthful, vigorous life.

